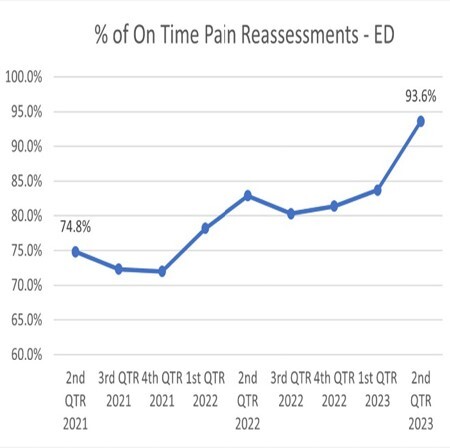# 552 Pain in the Assessment: Improving Reassessment of Burn Patients in the Emergency Department

**DOI:** 10.1093/jbcr/irae036.186

**Published:** 2024-04-17

**Authors:** Stacey Richerbach, Jenny Granger, Tiffany Hockenberry, Karen J Richey, Kevin N Foster

**Affiliations:** Arizona Burn Center Valleywise Health, Phoenix, AZ; Valleywise Health, Phoenix, AZ; Arizona Burn Center Valleywise Health, Phoenix, AZ; Valleywise Health, Phoenix, AZ; Arizona Burn Center Valleywise Health, Phoenix, AZ; Valleywise Health, Phoenix, AZ; Arizona Burn Center Valleywise Health, Phoenix, AZ; Valleywise Health, Phoenix, AZ; Arizona Burn Center Valleywise Health, Phoenix, AZ; Valleywise Health, Phoenix, AZ

## Abstract

**Introduction:**

Treatment of burn patients in the emergency department (ED) is challenging and often complicated by excruciating pain sparked by the burn injury. Unmanaged burn pain causes undue stress to the patient, both mentally and physically, and may result in physiologic manifestations. The physiologic impact of extreme burn pain may convolute a provider’s assessment and priorities of care. To facilitate appropriate treatment, RNs in the ED must perform timely pain reassessments. Our institutional benchmark for pain reassessment following medication administration is 90%. In 2021, we observed a drop in compliance in the ED to 73% and instituted a multidisciplinary, quality improvement (QI) approach to improve pain reassessment. The purposes of this project were to pinpoint education needs of nursing staff, improve compliance of pain reassessment, and to evaluate the efficacy of our interventions.

**Methods:**

A survey was disseminated to Burn ED nursing staff to identify barriers to pain reassessment. QI interventions included a collaboration between Nursing, Informatics, and Quality Assurance to overcome barriers related to education, optimization of documentation in the electronic health record (EHR), and timely performance feedback. Education was disseminated via pre-shift nursing huddle, weekly newsletters, and educational posters. Modifications were made to the EHR software to alert nurses to reassessment needs and accounted for medication ‘indication.’ Quality Assurance increased report frequency to furnish reports weekly instead of monthly, enabling nursing leadership to expedite individualized feedback and education. Feedback was well-received by staff. Reassessment metrics were added to the quality board and posted in staff lounges. Top performers were acknowledged monthly in the department newsletter’s ‘Benchmark Brief.’ Performance was analyzed from April 2021 through June 2023, and was grouped quarterly.

**Results:**

Following initiation of the QI project, pain reassessment increased by 18.8% to a 93.6% rate of timely reassessment.

**Conclusions:**

Our multidisciplinary quality improvement project was successful, increasing complete and timely pain reassessment in the ED by 18.8%. Further, we discovered the need to reinforce education related to medication administration route, as PO medications had a higher incidence of pain reassessment non-compliance. To reduce the limitations of staff education, we continue to work with Informatics and the EHR vendor to refine the use of technology and better facilitate reassessment.

**Applicability of Research to Practice:**

Further research is needed to evaluate efficacy of education related to pain reassessment following PO medication administration.